# Alternative Oxidase Expression in the Mouse Enables Bypassing Cytochrome *c* Oxidase Blockade and Limits Mitochondrial ROS Overproduction

**DOI:** 10.1371/journal.pgen.1003182

**Published:** 2013-01-03

**Authors:** Riyad El-Khoury, Eric Dufour, Malgorzata Rak, Nelina Ramanantsoa, Nicolas Grandchamp, Zsolt Csaba, Bertrand Duvillié, Paule Bénit, Jorge Gallego, Pierre Gressens, Chamsy Sarkis, Howard T. Jacobs, Pierre Rustin

**Affiliations:** 1 Inserm, UMR 676, Hôpital Robert Debré, Paris, France; 2 Faculté de Médecine Denis Diderot, Université Paris 7, Paris, France; 3 Institute of Biomedical Technology and Tampere University Hospital, Tampere, Finland; 4 NewVectys, Paris, France; 5 INSERM U845, Research Center Growth and Signaling, Hôpital Necker, Paris, France; 6 Faculté de Médecine, Université Paris Descartes, Paris, France; 7 Molecular Neurology Research Program, University of Helsinki, Helsinki, Finland; Max Planck Institute for Biology of Ageing, Germany

## Abstract

Cyanide-resistant non-phosphorylating respiration is known in mitochondria from plants, fungi, and microorganisms but is absent in mammals. It results from the activity of an alternative oxidase (AOX) that conveys electrons directly from the respiratory chain (RC) ubiquinol pool to oxygen. AOX thus provides a bypath that releases constraints on the cytochrome pathway and prevents the over-reduction of the ubiquinone pool, a major source of superoxide. RC dysfunctions and deleterious superoxide overproduction are recurrent themes in human pathologies, ranging from neurodegenerative diseases to cancer, and may be instrumental in ageing. Thus, preventing RC blockade and excess superoxide production by means of AOX should be of considerable interest. However, because of its energy-dissipating properties, AOX might produce deleterious effects of its own in mammals. Here we show that AOX can be safely expressed in the mouse (MitAOX), with major physiological parameters being unaffected. It neither disrupted the activity of other RC components nor decreased oxidative phosphorylation in isolated mitochondria. It conferred cyanide-resistance to mitochondrial substrate oxidation and decreased reactive oxygen species (ROS) production upon RC blockade. Accordingly, AOX expression was able to support cyanide-resistant respiration by intact organs and to afford prolonged protection against a lethal concentration of gaseous cyanide in whole animals. Taken together, these results indicate that AOX expression in the mouse is innocuous and permits to overcome a RC blockade, while reducing associated oxidative insult. Therefore, the MitAOX mice represent a valuable tool in order to investigate the ability of AOX to counteract the panoply of mitochondrial-inherited diseases originating from oxidative phosphorylation defects.

## Author Summary

In mammalian mitochondria, the energy-producing machinery is powered by the electron transfer to molecular oxygen, a mechanism whose terminal step is mediated by the cyanide-sensitive cytochrome *c* oxidase (COX). In plants, fungi, microorganisms, and some lower animals (but not in mammals), in addition to the normal pathway, a cyanide-resistant alternative oxidase (AOX) exists. It maintains electron transfer to oxygen even when the normal pathway is blocked. This provides a bypath that releases constraints on the energy producing machinery and prevents the production of deleterious superoxide molecules. Thus, preventing the energy producing machinery blockade and excess superoxide production by means of AOX should be of considerable interest. However, because of its energy-dissipating properties, AOX might produce deleterious effects of its own in mammals. Here we show that the AOX can be safely expressed in a mammal with major physiological and molecular parameters being unaffected. We also show that the AOX is active *in vivo* where it counteracts the energy producing machinery blockade and reduces *in vitro* the associated oxidative insult. Up to now, efficient therapies against mitochondrial-associated diseases are lacking dramatically. Therefore, in view of our results, the MitAOX mice represent a precious tool to assess the AOX therapeutic capacity against the panoply of inherited mitochondrial diseases.

## Introduction

**Figure 1:**
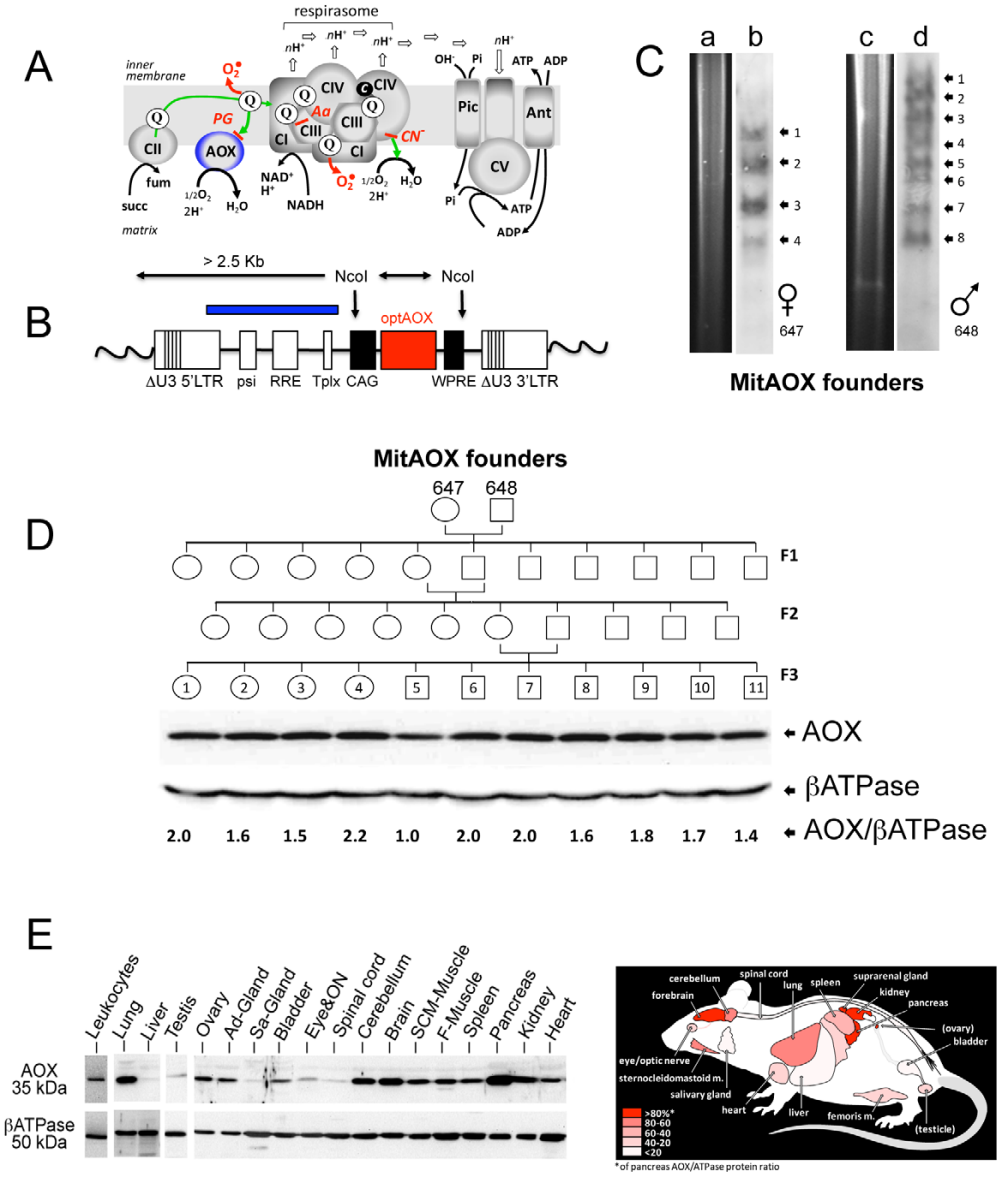
MitAOX mouse generation. A, an over simplified scheme of the respiratory chain featuring the alternative oxidase (AOX), inhibitable by propylgallate (PG). Electron flow (green) from dehydrogenases (Complex II, CII; Complex I, CI) converges to reduce complex III (CIII) ubiquinone (Q) pool, being channeled to oxygen through the respirasome, known to associate complexes I/III/IV in variable proportions. Over reduction of the Q pool activates the AOX that hinders any overproduction of superoxide (red). B, Lentiviral genome containing the AOX-expressing cassette, randomly integrated in the mouse genome (wavy lines). NcoI restriction enzyme was used to digest mouse tail genomic DNA. The blue box corresponds to the cassette fragment used as a probe. C, Ectopic integrations quantification by Southern blot (b, d) and digestion profiles (a, c) for the two founders (individuals 647 and 648) used to produce the 3 generations. D, Western blot analysis of F3 descendants from MitAOX founders 647, 648, showing the consistent AOX expression level in the brain with a 1 to 2.2 ratio to the ßATPase-subunit signal. E, Quantification of AOX protein levels, shown in the diagram, represents percentage of the expression in the highest expressing tissue (pancreas), based on signals from three MitAOX mice. F-Muscle, femoral muscle; SCM-Muscle, sternocleidomastoid muscle; ON, optic nerve; Sa-Gland, salivary gland; Ad-Gland, adrenal gland.

In mammalian mitochondria, the terminal step of electron transfer to molecular oxygen, producing water, is exclusively mediated by the cyanide-sensitive cytochrome *c* oxidase (COX) [1] and the electron transfer is tightly coupled to proton translocation. Protons simultaneously accumulated on the outer surface of the inner membrane are subsequently used by the ATP synthase (complex V, CV) to generate ATP from ADP and inorganic phosphate imported in the mitochondrial matrix by the adenylate carrier (Ant) and the phosphate carrier (Pic) respectively [2] (Figure 1A). Usually, a small percentage of electrons escapes from the RC to produce superoxide, with proposed roles in metabolic signaling [3]. However, conditions leading to the over-reduction of the ubiquinone pool may result in the production of excess superoxide, with deleterious consequences [4], [5]. In plants, many microorganisms, and a few animals [6], a non proton-motive, cyanide-resistant AOX, can also oxidize ubiquinol to produce water [7] (Figure 1A), maintaining electron transfer even when the activity of the cytochrome segment of the respiratory chain (namely complex III to IV) is limiting or unavailable [8]. Under such conditions, AOX also prevents the over-reduction of ubiquinone, serving, in effect, an antioxidant role [9]. Crucially, the enzymatic properties of AOX (low ubiquinol affinity) tend to limit its involvement in respiration *in vivo* to conditions of substantial over-reduction of the quinone pool, minimizing detrimental competition with the phosphorylating cytochrome pathway [10]. Nevertheless, in case of blockade of the cytochrome pathway, AOX enables divalent electron flow to oxygen, thus acting as a safety valve to preserve respiration, restore metabolic balance, and minimize excessive superoxide production [11]. Accordingly, we previously showed that *Ciona intestinalis* AOX could be expressed in cultured human cells, conferring cyanide-resistant respiration without harmful effects [12] and counteracting the consequences of genetic defects in COX [13]. Similarly, viable and active flies ubiquitously expressing AOX and substantially resistant to the action of antimycin (a complex III-specific inhibitor; Figure 1A) or cyanide were obtained [14]. AOX expression in flies also rescued the lethality of genetically-induced COX deficiency [14]. Altogether, these findings were an incentive to attempt AOX expression *in vivo* in a mammal.

Here we show that the AOX can be expressed safely in a mammal, without any obvious detrimental effect on major physiological parameters. We show also that the presence of the AOX conferred cyanide-resistance to mitochondrial substrate oxidation and decreased ROS production under conditions of RC inhibition. Importantly, the AOX also conferred cyanide-resistance to intact organ respiration and significantly prolonged the survival of the whole organism in the presence of this deadly poison. Taken together, these results indicate that the AOX is active *in vivo* in the MitAOX mouse and counteracts respiratory chain blockade and its physiological consequences.

## Results

**Figure 2:**
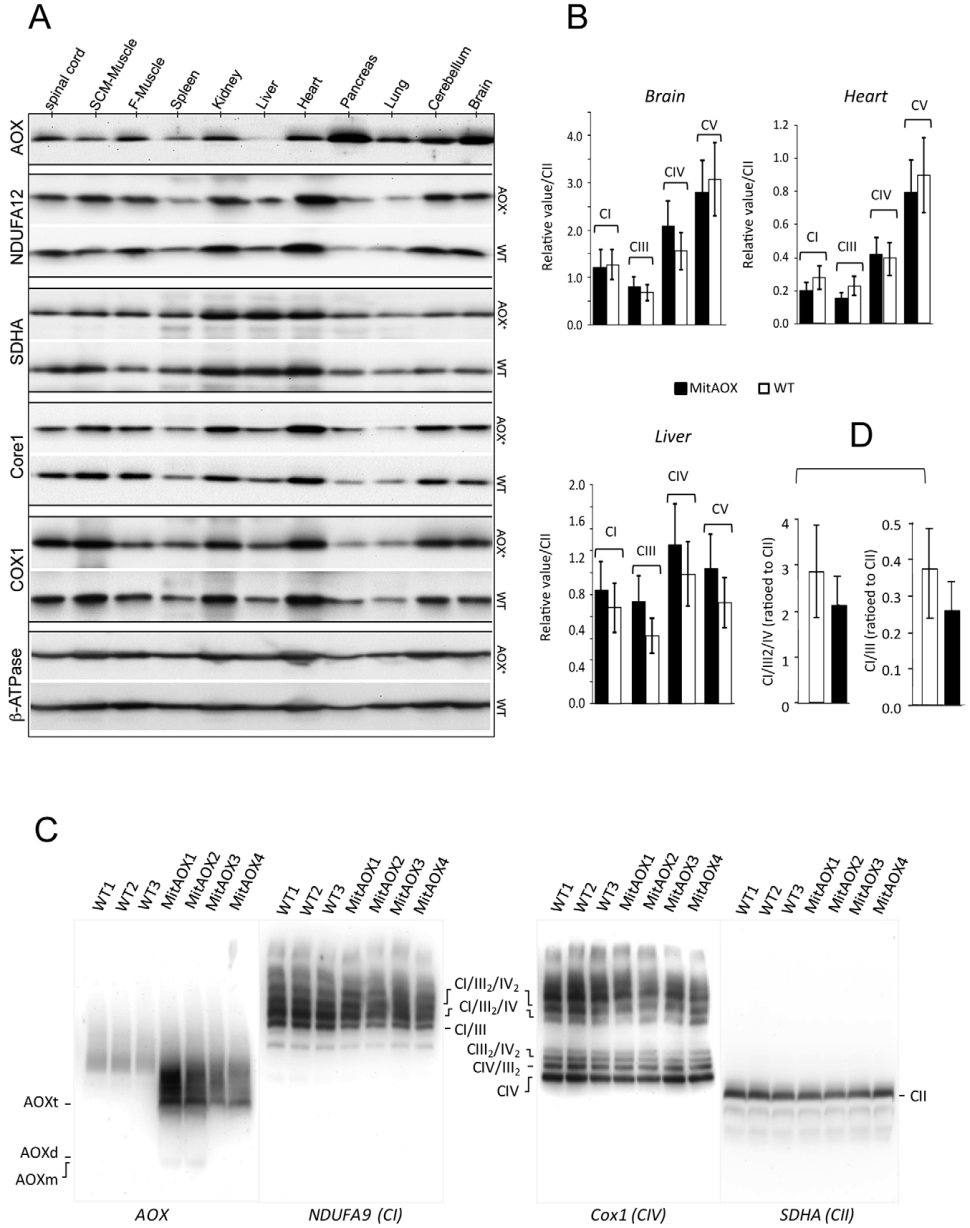
Western-blot and BN-PAGE analyses of brain mitochondria from WT and AOX mice. A, Western-blot analyses of the steady state of different RC complexes (CI, NDUFA12; CII, SDHA; CIII, Core1; CIV, Cox1 and, CV, ß-ATPase) of mitochondria extracted from 11 organs from four WT and four AOX mice. B, RC complexes (I, III, IV and V) from three different organs, highly (brain), mildly (heart) and weakly (liver) expressing the AOX, were quantified as a ratio to complex II with no significant difference being observed between WT and AOX mice. C, BN-PAGE analyses of cortex mitochondria stained with antibodies raised against AOX, NDUFA9, Cox1 and SDHA, under non-denaturating conditions (6 g/g digitonin). Supercomplex entities (three independent experiments) were found slightly but not significantly decreased in the MitAOX mice. D, Super-complexes partial quantification based on supercomplex (CI/III2/IV) band relative to that of CII.

The *Ciona intestinalis* AOX gene was recoded to maximize its expression in the mouse and introduced into early mouse embryos by germ-line lentiviral transduction on a mixed genetic background (CD-1/B6). We used the ubiquitously active, chimeric CAG promoter, together with the Woodchuck hepatitis virus Post-transcriptional Regulatory Element (WPRE) to further enhance AOX gene expression (Figure 1B). PCR analysis of genomic DNA up to the F3 generation indicated the presence of the AOX transgene in all founder descendants. Copy number was estimated by Southern blot at 4 to 8 per genome in the founders (Figure 1C). Western blot analysis of the F3 generation brain mitochondria indicated a consistent level of AOX protein between siblings (Figure 1D). Litter size (12±2 versus 11±3 in control mice) was unaffected by the presence of the transgene. A number of F3 individuals were analyzed for AOX distribution pattern. Western blots indicated, similarly to F1 individual (Figure 2A), widespread tissue expression, with expression prominent in brain and pancreas and varying among different tissues (Figure 1E). We also checked the stability of AOX expression as a function of age and observed a preserved AOX expression in all the different tissues studied in 15 month-old animals (Figure 2A). We next showed that the presence of the AOX did not alter the steady state levels of the different RC complexes (Figure 2A–2B). Noticeably, AOX did not require tight association with any of the RC complexes or supercomplexes in order to be functional, since the enzyme was not found associated with these entities in BN-PAGE analyses (Figure 2C). AOX migrated as a dimer with an apparent molecular weight of 70–72 kDa, with a substantial proportion found as higher polymeric forms, mostly tetramer, or tending to aggregate under this condition (6 g/g digitonin), as previously observed in organisms where AOX is naturally present [15]. Finally, we showed that neither the distribution nor the quantities of the RC supercomplexes were significantly modified by the presence of the AOX (Figure 2D).

**Figure 3:**
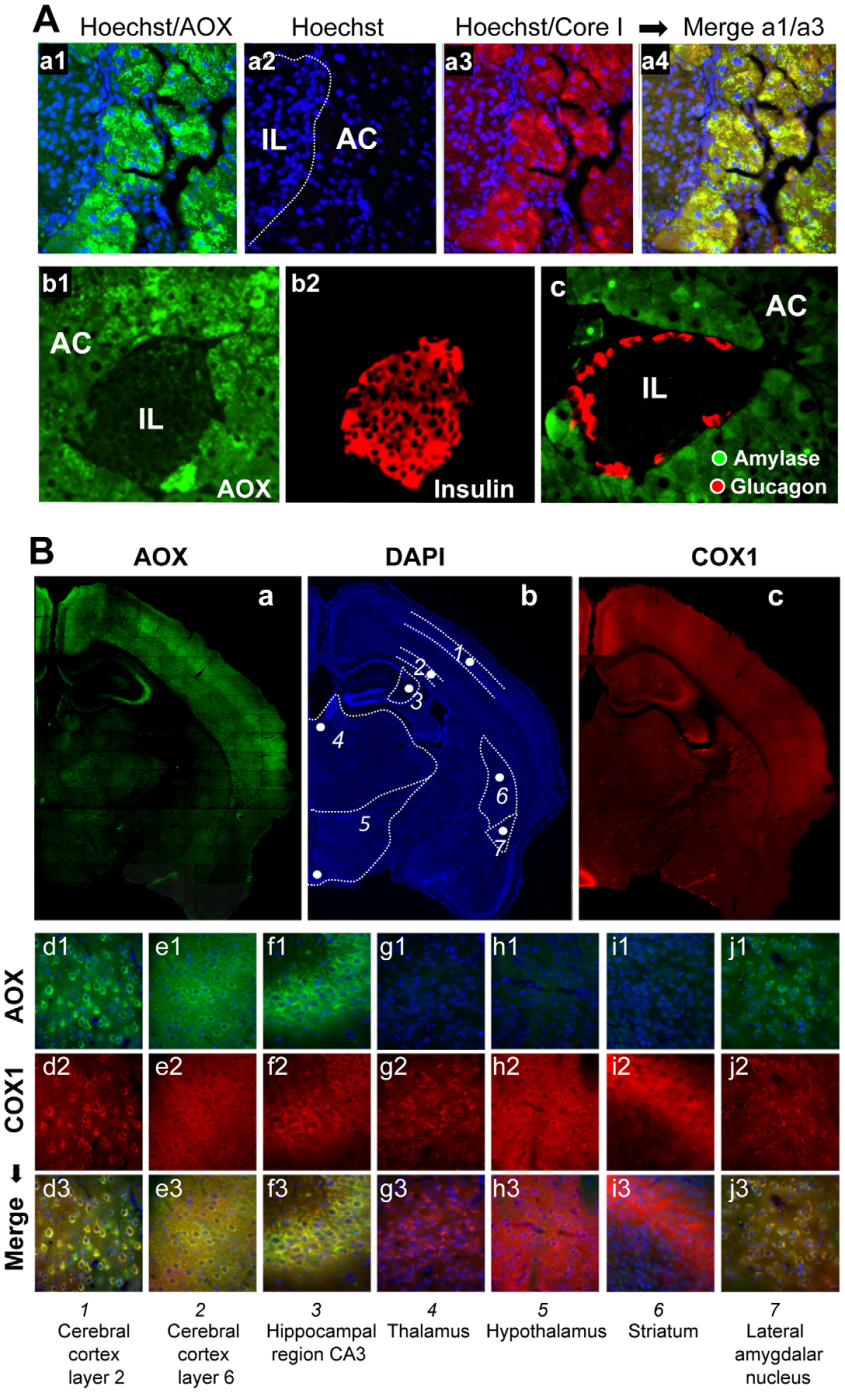
AOX expression territories in the pancreas and brain of the MitAOX mouse. A, Immunohistochemical study of MitAOX mouse pancreas sections showing AOX protein distribution (green; a1, b1) in Langerhans islets (IL) and acinar cells (AC). AOX (a1) is especially noticed in acinar cells where it perfectly overlaps with mitochondria stained by Core I antibody (red; a3). Despite the presence of numerous nuclei (a2) indicative of a high cell number, AOX and Core I antibodies stained only faintly Langerhans Islets. Noticeably, AOX pancreatic expression did not hamper insulin production by ß cells (b2), glucagon by α cells (c), or amylase in the exocrine pancreas (c). B, Immunohistochemical study of MitAOX brain showing region-specific distribution of AOX (a) independently of cell density estimated by nuclei staining (b) and only partially overlapping the distribution of mitochondria, stained with COX I antibody (c). Analyses (d–j) of brain regions (numbered according to b). AOX staining (d1–j1; green) overlaps perfectly with COX I staining (d2–j2; red) in the cerebral cortex layer 2 and 6 (L2, L6), in the hippocampal region CA3 and in the lateral amygdalar nucleus (d3–e3, j3; yellow). No AOX protein was convincingly detected in the thalamus, hypothalamus or striatum (g–i).

Detailed immunohistological analysis of highly AOX-expressing tissues revealed differential expression depending on organ sub-territories. For instance, in the pancreas, the pattern of AOX expression matched that of complex III Core protein I (Core I) and COX I (complex IV; not shown), being much higher in exocrine than in endocrine tissue (insulin-producing Langerhans islets; Figure 3A). In the brain (Figure 3B), AOX was massively expressed in the CA3 pyramidal layer and the cortex, with a perfect overlap with COX I (or Core I, or ATPase α (complex V); not shown) antibody staining. AOX expression was lower in the lateral amygdalar nucleus, even lower in the CA1 pyramidal layer, and hardly detectable in the thalamus, the hypothalamus, or the granule cell layer of the dentate gyrus, despite strong staining with COX I (Figure 3B) or other OXPHOS marker antibodies (Core I, ATPase α; not shown). Interestingly, immunohistological study performed on the brain of WT and MitAOX mice using COX I antibody revealed similar expression, which denotes the absence of detectable effect of AOX expression on the amount and distribution of mitochondria in the brain (Figure S1).

**Figure 4:**
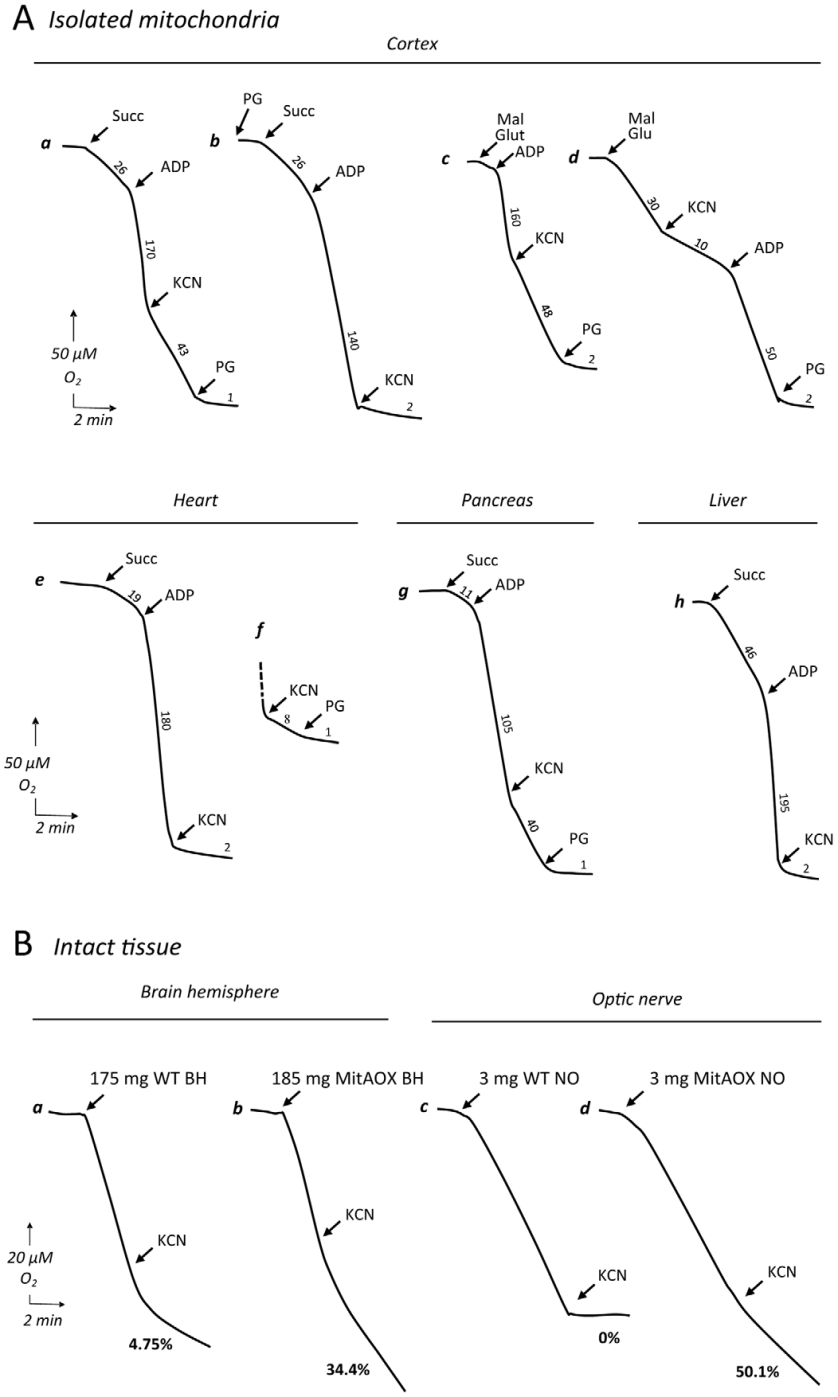
Polarographic analysis of oxygen uptake in isolated mitochondria and intact organs. A, Mitochondrial substrate (succinate or malate+glutamate) oxidation was strongly stimulated by ADP in mitochondria from all investigated tissues (a–h). A significant cyanide-resistant oxidation was observed in brain (a–c) and pancreas (g) mitochondria of the MitAOX mouse and was fully inhibited by PG (a, c, d). Finally, a limited yet significant cyanide-resistant succinate oxidation was measured in heart using 3 times more mitochondria (f). Cyanide-insensitive, PG-sensitive substrate oxidation was never observed in wild-type mitochondria (not shown). Numbers along the traces are means of three independent replicates and refer to nmol/min/mg protein. Mitochondrial protein: 100 to 150 µg. B, forebrain cerebral hemisphere (a, b) and optic nerve (c, d) respiration from WT (a, c) and MitAOX (b, d) mice. Intact forebrain hemisphere was suspended in 1.5 ml of respiratory medium in the Clark oxygen electrode chamber using a homemade device supporting a nylon net (200 µm mesh). Whole optic nerve was placed directly in the electrode chamber in 250 µl of respiratory medium. Both brain and optic nerve respiration was found linear for more than ½ hour in the respiratory medium. Noticeably, unspecific inhibitory effect of PG on WT and MitAOX tissue respiration (full inhibition of WT brain respiration) precluded the use of this inhibitor to study tissue respiration.

Mitochondria were next isolated from a number of tissues as to investigate AOX functionality. A significant cyanide-resistant oxidation of succinate was detected in several tissues and was proportional to the AOX protein level. For instance, tissues with the highest protein expression (brain and pancreas), showed the highest cyanide resistance, which was less in heart and nearly inexistent in liver (Figure 4A, traces a, c, e, g, h) with the lowest protein level. In all cases, cyanide resistant respiration was fully inhibited by 50 µM propylgallate (PG), a specific inhibitor of AOX. A quite similar cyanide-resistance (about 30%) was measured using malate *plus* glutamate as substrate (Figure 4A, trace c). Noticeably, the oxidation of malate *plus* glutamate was still efficiently controlled by the phosphorylation process in the MitAOX mouse. This is shown by the large stimulation of malate oxidation triggered by the ADP addition in the presence of cyanide (Figure 4A, trace d).

Because AOX expression in the brain or in the pancreas (Figure 3) was not uniform, the true extent of cyanide resistance in AOX-expressing sub-territories in these tissues is presumably even higher. In comparison, whatever the substrate being oxidized (succinate or malate) by mitochondria of WT animal tissues, cyanide fully inhibited oxygen consumption (less <1% resistance to cyanide; Table S1). Afterwards, using a homemade device supporting a nylon net, we were able to study the whole organ respiration using the Clark oxygen electrode chamber. As compared to WT, a significant cyanide-resistance of whole organ respiration, 30% and 50% for brain hemisphere and optic nerve respectively (Figure 4B, traces a–d) was observed in MitAOX.

**Figure 5:**
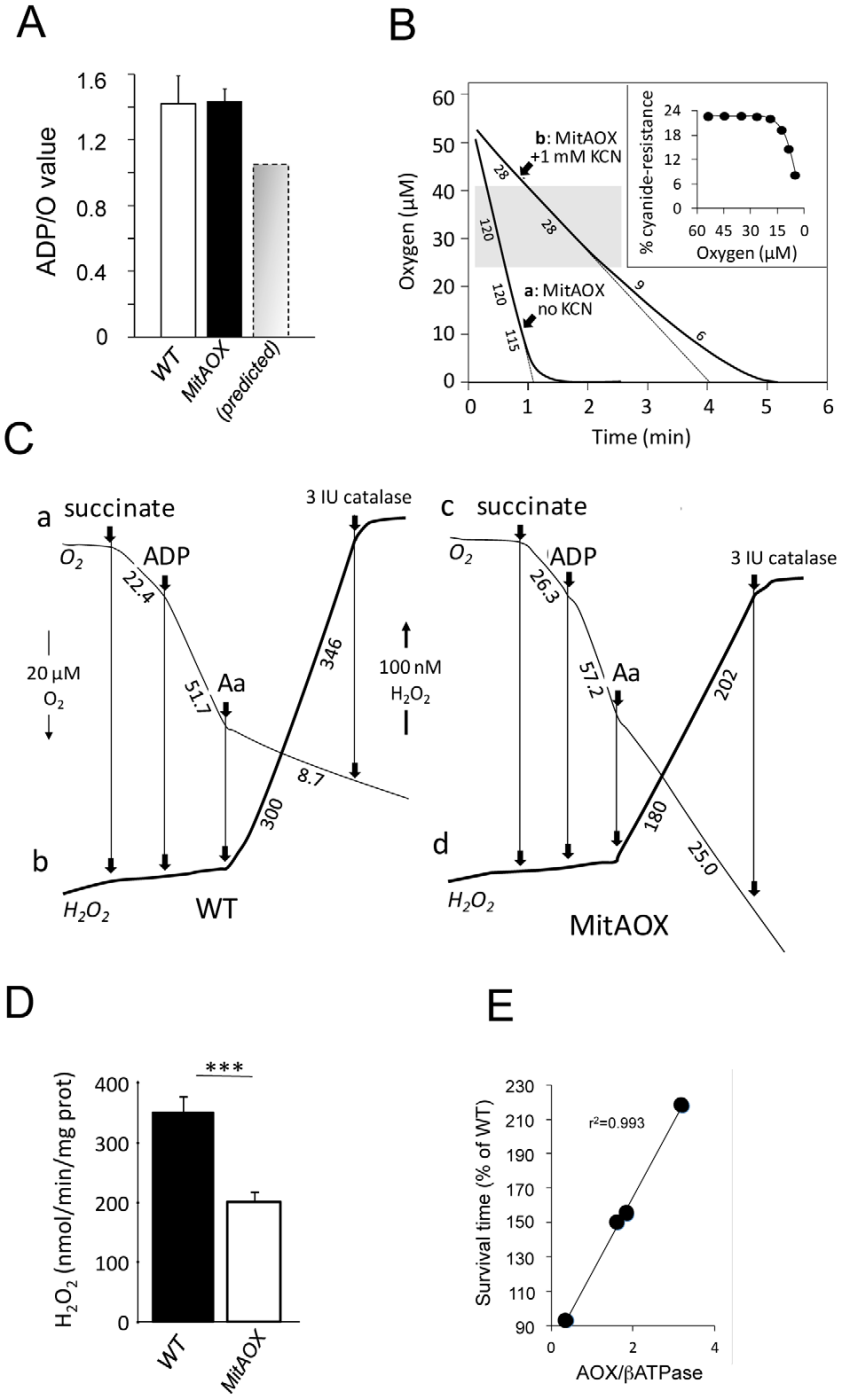
Consequences of AOX expression on mitochondrial properties in the MitAOX mouse. A, ADP/O values in cortex mitochondria oxidizing succinate. Predicted value (grey) estimated for a 30% cyanide-resistant succinate oxidation (Figure 4A) and full operation of the AOX. B, Oxygen uptake by 100 µg cortex mitochondria oxidizing 10 mM succinate in the presence of 500 µM ADP, in the absence (a) or presence (b) of KCN. Numbers along the traces are nmol/min/mg protein Inset: cyanide-resistance plotted *versus* oxygen concentration. C, Simultaneous measurement of oxygen consumption (a, c) and reduction of the Amplex Red dye (b, d) by 300 µg cortex mitochondria oxidizing succinate as described under material and methods. Numbers along traces refer to oxygen uptake (a, c) or hydrogen peroxide (b, d) production. D, bar graph indicating the mean value (four independent experiments) ± SD of H_2_O_2_ production by WT and MitAOX brain mitochondria oxidizing succinate in the presence of antimycin as in C. E, Anesthetized MitAOX and WT mice were exposed to cyanide and survival time noted. Expressed as a percent of WT mice survival time, data for each MitAOX mouse tested were plotted as a function of AOX/ATPase protein levels in brain and lungs, quantitated from Western blots.

In order to estimate indirectly the participation of AOX in the oxidation of succinate under phosphorylating conditions (presence of ADP) we determined the ADP/O values in WT and MitAOX mice brain mitochondria (Figure 5A). Any significant involvement of the non-proton motive AOX in electron flow should decrease the use of ADP associated with O_2_ consumption (Figure 1A), thereby diminishing the ADP/O ratio. The measured values (≈1.4; Figure 5A) were found to be similar in MitAOX and WT brain mitochondria, indicative of a negligible participation of AOX in electron transfer in the presence of ADP, as previously reported for mitochondria of organisms naturally endowed with AOX [16]. Extensive investigations of brain and pancreas mitochondria revealed no significant impact of AOX expression on RC activities (Figure S2A). Because the low O_2_ level *in vivo* (*i.e.* 25 to 40 µM in the brain) might affect the activity of the AOX, we next tested cyanide-resistance of substrate oxidation as a function of O_2_ concentration (Figure 5B). We observed a gradual decrease of cyanide-resistance only for O_2_ concentrations below 20 µM. At these low oxygen tension values, percent of cyanide-resistance was confirmed by the simultaneous measurement of oxygen consumption in a closed chamber by a classical Clark electrode and a fluorescence-based micro-optode (Figure S2B, S2C). AOX thus appears to be fully functional under physiological conditions. Interestingly, mitochondria from MitAOX mouse brain produced significantly less ROS than their WT counterparts, despite partial expression in the brain (Figure 3B). This was shown by an assay in which superoxide, whose production was triggered by antimycin, was fully converted to hydrogen peroxide (Figure 5C–5D). Noticeably before the addition of antimycin, the limited production of ROS is not affected by the presence of the AOX.

In order to evaluate a potential detrimental effect of AOX expression, we next investigated a number of physiological and behavioral variables in pups and mature MitAOX animals (Table 1). Cardiorespiratory variables displayed minor, albeit statistically significant differences between MitAOX and WT newborn mice. In addition to their slightly smaller weights (<10%), the MitAOX pups had slightly slower heart rates (<8%) while minor differences in their breathing pattern did not affect ventilation. Activity and ultrasonic vocalizations (a common marker of anxiety in newborn rodents) showed no significant genotype-related differences. Thus, the analysis of physiological and behavioral variables in MitAOX newborn mice under normal conditions did not reveal any pathological signs. Later on, at 3 months of age, MitAOX animals had similar weight and performed as WT in the Rotarod test, documenting unaltered motor coordination and fatigue resistance (Table 1), despite significant AOX expression in the cerebellum and skeletal muscle (Figure 1E).

**Table 1:** Physiological variables in MitAOX and wild-type mice.

*Parameter*	*MitAOX mice*	*WT mice*	*p-value*
*6 days of age* *			
Weight (g)	3.32±0.41	3.65±0.36	0.0067
Body temperature (°C)	36.6±0.3	36.3±0.1	0.0037
Activity duration (% time)	8.11±9.08	9.62±9.66	0.5954
Heart rate (beats min^−1^)	483±36	521±47	0.0181
Ventilation (µl.s^−1^.g^−1^)	25.32±4.05	23.56±4.93	0.2006
Tidal volume (µl.g^−1^)	7.66±1.11	6.60±1.23	0.0047
Breathing rate (breaths min^−1^)	199±31	214±23	0.0774
Ultrasonic vocalisations (s)	0.57±1.61	0.26±0.48	0.4121
*2 months of age*			
Weight (g): females (n = 9)	28.3±4.1	26.6±3.6	0.1444
Weight (g): males (n = 8)	36.3±3.8	32.3±3.6	0.1252
Latency to fall on the rotarod (s) (n = 12)	143±21	139±56	0.8001

* At 6 days of age, cardiorespiratory variables of MitAOX (n = 23) and WT mice (n = 21) were averaged over a 10 min period at 33°C. Activity duration is expressed as percentage of total recording period (10 min). Duration of ultrasonic vocalisations was totalized over 10 min. Values are group means (±SD).

Finally, in order to assess the capacity of the AOX to compensate for a cytochrome pathway blockade *in vivo*, anesthetized MitAOX mice were exposed to gaseous cyanide. The deadly effect of cyanide on mammals has been previously shown to result mostly from the inhibition of the mitochondrial cytochrome oxidase rather than to its binding to other metalloenzymes [17]. Accordingly, we observed a substantially prolonged survival of MitAOX mice in the presence of a lethal concentration of gaseous cyanide, compared to WT mice (more than 200%). Moreover, by using different transgenic founders with variable AOX expression, we were able to show that the amount of resistance to cyanide in the whole animal is proportional to AOX protein content as determined in the lung and in the brain (Figure 5E).

## Discussion

Our data show for the first time that a functional AOX can be expressed in a mammal and transmitted between generations, conferring significant cyanide-resistance to mitochondrial substrate oxidation and tissue respiration as well as the whole organism. As previously observed in cultured human cells [12], [18] and flies [14], [19], the enzyme is targeted to the mitochondria where it functionally interacts with the RC. Most importantly, our data show that, similarly to the plant enzyme [8], the *C. intestinalis* AOX expressed in the MitAOX does not interfere/compete significantly with the cytochrome pathway, being functional only upon blockade of this latter when the pool of ubiquinone becomes highly reduced. Accordingly, the expression of the *C. intestinalis* AOX in the mouse did not result in any deleterious consequence, whilst spectacularly increasing the survival of the mouse in the presence of a lethal concentration of gaseous cyanide. The protective mechanism provided by the AOX to organisms naturally harboring the enzyme was therefore fully preserved when the oxidase was expressed in the mouse.

The AOX protein typically has such a high Km for reduced quinones (apparent Km_DQH2_ from 0.53 to 0.38 mM) [20] that it competes only very poorly with the cytochrome pathway (apparent Km_DQH2_ less than 20 µM) [21] for quinol oxidation. In plants, such a competition is further avoided by a specific channeling of electrons, dictated by the structural association of the relevant electron carriers, involving the malic enzyme, the Ndi (internal rotenone-insensitive NADH dehydrogenase), and the AOX proteins [22], [23]. Noticeably, in the absence of Ndi in the MitAOX mouse, the non proton-motive AOX can still promote ATP formation through activation of NADH oxidation by the proton-motive complex I.

The control of superoxide overproduction by AOX [24], [25] illustrates a second protective effect resulting from the AOX expression in the mouse. According to our data, *C intestinalis* AOX efficiently decreases superoxide overproduction triggered by the over-reduction of the ubiquinone pool, which was generated by antimycin. On the other hand, the innocuousness of AOX expression in the mouse suggests that any production of superoxide that is physiologically required [26], [27] is not significantly modified by the presence of a functional AOX in the mitochondria.

The observed protection provided by the AOX against the toxic effects of cyanide or antimycin, in the light of its expression territories, should enable the use of the MitAOX mouse to testing the potentially deleterious role of mitochondrial dysfunction and/or the resulting oxidative stress in mouse models of neurodegenerative diseases [28]. It will be similarly interesting to use the MitAOX mouse to investigate the potential role of mitochondrial dysfunction and excess ROS production in ageing [29]. Indeed, a single polypeptide, AOX, can replace two elaborate multisubunit complexes (complexes III and IV; 11 and 13 subunits respectively), without competing with these under normal conditions. AOX expression may thus also chart a way to implementing a wide-spectrum therapy for currently intractable but major disease entities [11].

Finally, we may wonder why the AOX, with the huge metabolic flexibility that it confers to the cell, has been lost in most of the animal kingdom. One intriguing clue comes from the fact that organisms naturally endowed with AOX are almost exclusively sessile or pelagic, and their mitochondria must regularly endure harsh energetic and stress conditions from which they cannot escape: *e.g.*, activation of photosynthesis for plants [30], [31], exposure to toxic xenobiotics for microorganisms, or local fluctuations in the marine environment (temperature, oxygen, nutrient levels) for animals that are fixed in one place [6]. In contrast, in fast-moving organisms, AOX activity would only be advantageous under peculiar conditions, such as those arising in cases of mitochondrial diseases where OXPHOS is primarily or secondarily affected. The creation of a mammal expressing the AOX will surely be crucial in shedding light on this puzzling evolutionary interrogation.

## Materials and Methods

### Animals

The mice were housed with a 12-h light/dark cycle and free access to food (3% lipids, 16% protein; SAFE A-04 chow; UAR Epinay sur Orge, France) and water. Animal management was in accordance with Good Laboratory Practice Guidelines [32]. All experiments were carried out following the recommendation of INSERM for the use of animal laboratory and the approval by the ethical committee of Debre-Bichat Hospitals; project number 2010-13/676-0014.

### Construction of the AOX-Expressing Vector

The *Ciona intestinalis* AOX cDNA sequence [12] was redesigned, optimized (AOXopt) in order to ameliorate its expression in mice (DNA2.0 algorithm) and synthesized by DNA2.0 (Menlo Park, CA, USA). The redesigned cDNA was flanked by the attL1/L2 recombination sites. Next, using Gateway cloning technology, the AOXopt cDNA was transferred by an *in vitro* one-step recombination to the attR1/R2-containing plasmid pTrip [33], further used to produce the AOX lentiviral vectors. Afterwards, the strong chimeric CAG promoter was isolated as a 1.7 kb digestion fragment (EcorV-Mlu1) from the p97 Vector and cloned upstream the AOX in the pTrip-AOXopt vector, giving rise to pTrip-CAG-AOXopt. Finally, the Woodchuck Hepatitis post-transcriptional Regulatory Element (WPRE) was isolated as a 650 bp digestion fragment (BstX1-Kpn1) from plasmid pT45 and introduced downstream of the AOXopt cDNA, giving rise to the pTrip-CAG-AOXopt-WPRE plasmid.

### AOX Lentiviral Vector Production and Purification

Lentiviral vectors containing the CAG-AOXopt-WPRE construct were generated as previously described [33]. Before injection, the HIV p24 Gag antigen was quantified by ELISA (HIV-1 P24 antigen assay; ZeptoMetrix corporation, NY, USA), and the AOX-expressing lentiviral vectors were titered by transducing 40,000 HeLa cells in 24-well plates with serial dilution (2, 1, 0.5 µl). Each early mouse embryo was injected with 50–500 pL (p24 vector titer: 109 ng/µl).

### Genomic DNA Analysis

Mouse genomic DNA was extracted from frozen tail samples using the MasterPure DNA Purification Kit according to the manufacturer's instructions (Tebu-Bio, Le Perray en Yveline, France). The transmission of the AOX transgene was verified by PCR on tail genomic DNA using AOXopt-F (GGATGAGCCCAATATCGAAG) and AOXopt-R (CTGAAACGAAAATGCCTTGG) primers. For Southern blot analysis the DIG System from Roche Applied Science was used.

### Protein Analysis

Western blot analyses were performed as indicated in [13]. In addition, blots were re-probed with an anti-ßATPase (1∶5000, rabbit polyclonal antibody raised against the yeast ßATPase and kindly provided by A. Tzagoloff) as a standardization control. Peroxidase-conjugated anti-rabbit secondary antibody (1∶5,000, Amersham, Buckinghamshire, UK) was used at 5,000-fold dilution. Blue-native PAGE (BN-PAGE) analyses were performed on isolated mitochondria as described [34]. Concentration of detergents as indicated in the figure legend.

### Enzyme Activities

Respiratory chain enzyme activities were spectrophotometrically measured using a Cary 50 UV–visible spectrophotometer (Varian Inc, Les Ulis, France) [35]. Mitochondrial substrate oxidation was polarographically estimated using a Clark oxygen electrode (Hansatech Instruments, Norfolk, England) in a magnetically-stirred chamber maintained at 37°C in 250 µl of a respiratory medium consisting of 0.3 M mannitol, 5 mM KCl, 5 mM MgCl_2_, 10 mM phosphate buffer (pH 7.2) and 1 mg.ml^−1^ bovine serum albumin, plus substrates or inhibitors as described [36]. Substrate and inhibitor concentrations were as followed: 1 mM ADP, 10 mM succinate, malate/glutamate (5 mM each), 1 mM KCN, 50 µM PG, 4 µM rotenone. Alternatively, oxygen uptake was measured under similar condition using a micro-optode consisting in an optic fiber equipped with an oxygen-sensitive fluorescent terminal sensor (FireSting O_2_; Bionef, Paris, France). Superoxide *plus* hydrogen peroxide production by isolated brain mitochondria was quantified using Amplex Red fluorescence in 1.5 ml of medium consisting of 125 mM KCl, 14 mM NaCl, 1 mM MgCl_2_, 20 µM EGTA, 4 mM KH_2_PO_4_ and 20 mM HEPES (pH 7.2) to which were added 2 IU of purified superoxide dismutase [37]. Inhibitory effect of propylgallate could not be tested in these assays because of interactions with the probe. Protein concentration was measured according to the Bradford assay.

### Immunohistochemistry

#### Brain analysis

Three-month-old mice were deeply anesthetized with chloral hydrate (150 mg/kg *i.p.*) and perfused through the ascending aorta with 20 ml of saline followed by 100 ml of 4% paraformaldehyde in 0.1 M phosphate buffer, pH 7.4 (PB). Brains were post-fixed overnight in 4% paraformaldehyde at 4°C, cryoprotected in 30% sucrose in PB (4°C, 24 h) and frozen in liquid isopentane at −45°C. Rabbit polyclonal anti-AOX antibody (1∶300), mouse monoclonal anti-Core1 (Core1; 1∶300; Fisher Scientific), and mouse anti-complex IV subunit I antibody (COX I; 1∶300; MitoSciences, Eugene OR, USA) were used for double-labeling fluorescent immunohistochemistry and proceeded as in [38].

#### Pancreas analysis

For pancreases, adult AOX transgenic mice and WT mice were dissected and fixed in 10% formalin. Sections (4 µm thickness) were collected and processed for immunohistochemistry as described previously [39]. The antibodies were used at the following dilutions: rabbit anti-AOX antibody (1∶500), mouse anti-Core 1 (1∶500), rabbit anti-COX I (1∶500). The fluorescent secondary antibodies were Alexa 488-conjugated goat anti-rabbit IgG (1∶400) and Alexa 633-conugated goat anti-mouse IgG (1∶400) antibody (Fisher Scientific). Photographs were taken using a fluorescence microscope Leica Leitz DMRB (Leica Microsystemes, Nanterre, France) and a Hamamatsu C5810 cooled 3CCD camera (Hamamatsu Photonics, Hamamatsu City, Japan). No signals were observed when the primary antibodies were omitted.

### Physiology

Breathing variables (breath duration (T_TOT_), tidal volume (V_T_), and ventilation (V_E_) calculated as V_T_/T_TOT_) were measured noninvasively in unanaesthetized, unrestrained 6-day old pups using whole-body flow barometric plethysmography as described previously [40], [41]. Statistical analyses were performed using Student's *t*-test (Statview 5). Values of p<0.05 were considered as significant. Motor coordination and fatigue resistance of older animals (2 months) were assessed by Rotarod test as previously described [42].

### Mice Exposure to Inhaled Cyanide

Cyanide poisoning is classified as an USDA Pain and Distress Category E condition, and the investigators estimated the study acceptable only if the animals were beforehand anesthetized. The investigators realized this might have impacted the outcome of the experiment, but that without the use of anesthesia, the work would have been inhumane. Mice were anesthetized with chloral hydrate (350 mg/kg). Once anesthetized, one WT and one MitAOX mouse were placed in a 5.2 L airtight acrylic glass chamber maintained at 28°C (above the boiling point of cyanide, 26°C). Cyanide gas (451 ppm) was produced in the chamber by injecting 100 mM KCN into a *Petri* dish containing 10 ml of 1 M sulfuric acid. Respiratory activity of the mice was used as an index of mouse survival. Four experiments were carried out with MitAOX mice endowed with different levels of AOX, afterwards estimated in the lung and the brain by Western blot analysis.

## Supporting Information

Figure S1 Cox1 distribution profile in the MitAOX and WT mice. Immunohistochemical study of MitAOX and WT brain stained with COX I antibody showing mitochondrial distribution.
(TIF)

Figure S2 Respiratory chain activities and oxygen consumption by WT and MitAOX mitochondria. A, Respiratory chain enzyme activities from MitAOX and WT mice brain homogenates were spectrophotometrically measured as described in materials and methods. B, Oxygen consumption and cyanide resistance of succinate oxidation by brain mitochondria from WT and MitAOX mice measured using a standard Clark electrode (a) or using a fluorescence-based micro-optode (b) as described under material and methods. C, Effect of cyanide as a function of oxygen tension under similar conditions measured with the micro-optode. For the sake of comparison, slopes were adjusted by modulating mitochondrial protein used to record oxygen consumption, allowing an easier comparison of the effect of low oxygen tension.
(TIF)

Table S1 Polarographic determination of substrate oxidation rates and cyanide resistance by brain or pancreas mitochondria. Experimental conditions as described in Figure 4.
(DOCX)
