# Supplementary figures and images for: Alternative Oxidase Expression in the Mouse Enables Bypassing Cytochrome c Oxidase Blockade and Limits Mitochondrial ROS Overproduction
Source: PLoS Genet. 2013 Jan 3;9(1):e1003182. doi: 10.1371/journal.pgen.1003182 (PMC3536694; doi:10.1371/journal.pgen.1003182)

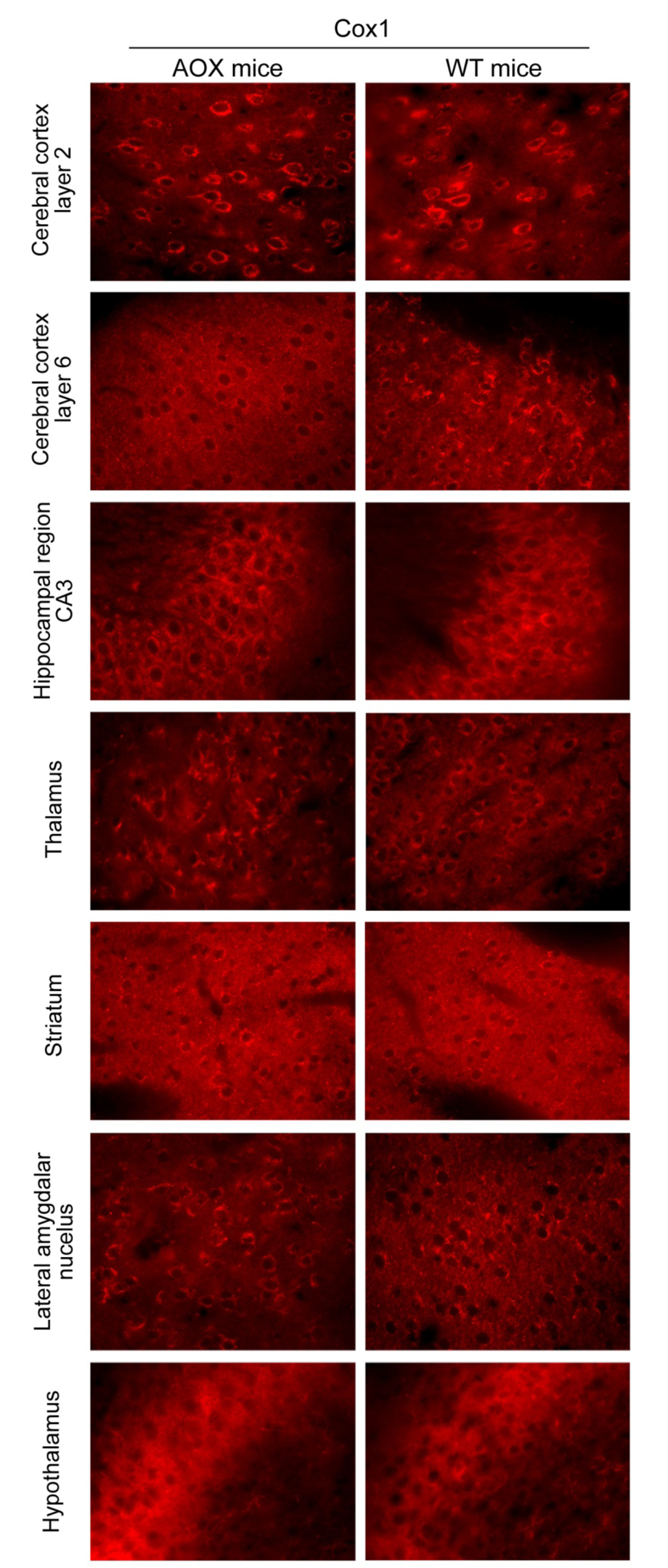

Supplement: Figure S1 — Cox1 distribution profile in the MitAOX and WT mice. Immunohistochemical study of MitAOX and WT brain stained with COX I antibody showing mitochondrial distribution. (TIF) [file pgen.1003182.s001.tif]

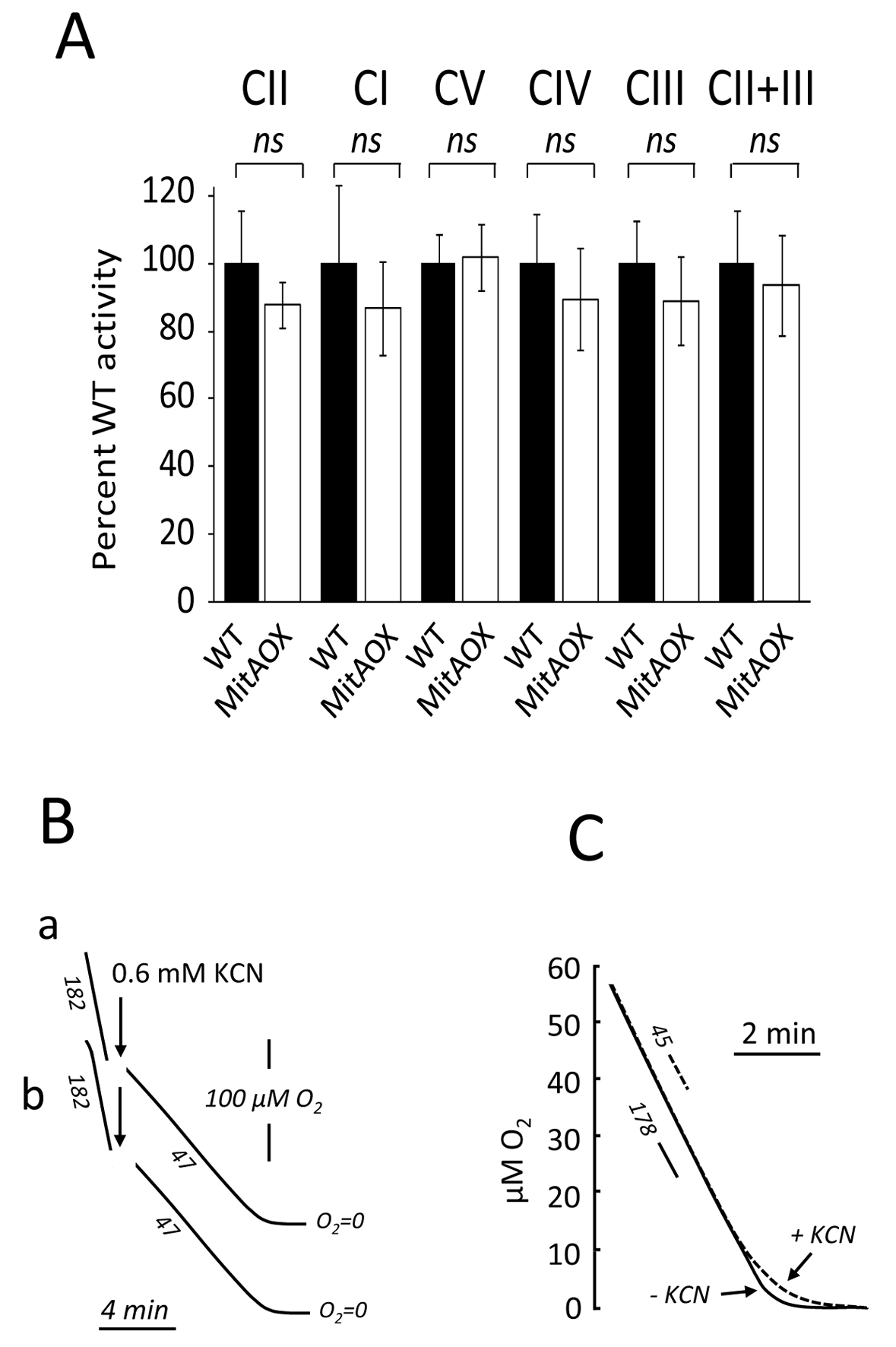

Supplement: Figure S2 — Respiratory chain activities and oxygen consumption by WT and MitAOX mitochondria. A, Respiratory chain enzyme activities from MitAOX and WT mice brain homogenates were spectrophotometrically measured as described in materials and methods. B, Oxygen consumption and cyanide resistance of succinate oxidation by brain mitochondria from WT and MitAOX mice measured using a standard Clark electrode (a) or using a fluorescence-based micro-optode (b) as described under material and methods. C, Effect of cyanide as a function of oxygen tension under similar conditions measured with the micro-optode. For the sake of comparison, slopes were adjusted by modulating mitochondrial protein used to record oxygen consumption, allowing an easier comparison of the effect of low oxygen tension. (TIF) [file pgen.1003182.s002.tif]
