# Supplementary material for: Alternative Oxidase Expression in the Mouse Enables Bypassing Cytochrome c Oxidase Blockade and Limits Mitochondrial ROS Overproduction
Source: PLoS Genet. 2013 Jan 3;9(1):e1003182. doi: 10.1371/journal.pgen.1003182 (PMC3536694; doi:10.1371/journal.pgen.1003182)
Supplement: Table S1 — Polarographic determination of substrate oxidation rates and cyanide resistance by brain or pancreas mitochondria. Experimental conditions as described in Figure 4. (DOCX) [file pgen.1003182.s003.docx]

| Substrate | Oxgen uptake  (nmol/min/mg prot) | | | Cyanide resistance  (% of state 3 rate) |
| --- | --- | --- | --- | --- |
| Succinate |  | KCN | propylgallate^1^ |  |
| *Brain WT* | 154±22 | 0.7±0.1 | - | <1 |
| *Brain MitAOX* | 158±25 | 40±4 | 3.9±0.4 | 25.7 |
| *Pancreas WT* | 101±8 | 0.6±0.1 | - | <1 |
| *Pancreas MitAOX* | 109±9 | 39±4 | 2.3±0.3 | 36.0 |
|  |  |  |  |  |
| Malate/Glutamate |  |  |  |  |
| *Brain WT* | 152±30 | 0.8±0.1 | - | <1 |
| *Brain MitAOX* | 160±13 | 48±5 | 1.6±0.1 | 29.9 |

^1^Propylgallate (+ 0.5 mM) was added subsequently to KCN (1 mM) addition
